# Stability-Indicating HPLC Method for the Simultaneous Determination of HIV Tablet Containing Emtricitabine, Tenofovir Disoproxil Fumarate, and Rilpivirine Hydrochloride in Pharmaceutical Dosage Forms

**DOI:** 10.1155/2014/849149

**Published:** 2014-10-29

**Authors:** S. Venkatesan, N. Kannappan, Sai Sandeep Mannemala

**Affiliations:** Department of Pharmacy, Annamalai University, Chidambaram, Tamil Nadu 608002, India

## Abstract

A simple, accurate, rapid, and stability-indicating RP-HPLC method for a combination of tenofovir disoproxil fumarate, emtricitabine, and rilpivirine has been developed and subsequently validated in commercial tablets. The proposed HPLC method utilizes Phenomenex Gemini C18 column (150 mm × 4.6 mm i.d., 5 *µ*m) and mobile phase consisting of MeCN, potassium dihydrogen phosphate buffer (20 mM, pH 3.3), and triethylamine 58.72 : 41.23 : 0.05 (v/v) at a flow rate of 1.7 mL/min. Quantitation was achieved with UV detection at 270 nm. The method was validated in terms of accuracy, precision, linearity, limits of detection, limits of quantitation, and robustness. This optimized method has been successively applied to pharmaceutical formulation and no interference from the tablet excipients was found. TDF, EMT, and RPV and their combination drug product were subjected to acid, base, neutral hydrolysis, oxidation, dry heat, and photolytic stress conditions and the stressed samples were analyzed by the proposed method. As the proposed LC method could effectively separate the drugs from its degradation products, it can be employed as stability-indicating method for the determination of instability of these drugs in bulk and commercial tablets.

## 1. Introduction 

Safety and efficacy of pharmaceuticals are two basic problems with importance in drug therapy. Instability of pharmaceuticals can cause a change in physical, chemical, pharmacological, and toxicological properties of the active pharmaceutical ingredients (API), thereby affecting its safety and efficacy. Hence, the pharmacists should take cognizance of various factors such as drug stability, possible degradation products, mechanisms and routes of degradation, and potential interactions with excipients utilized in the formulation to ensure the delivery of their therapeutic values to patients. In order to assess the stability of a drug product, one needs an appropriate analytical methodology, the so-called stability-indicating methods which allow accurate and precise quantitation of the drug and its degradation products and interaction products, if any. In recent times, the development of stability-indicating assays has increased dramatically using the approach of stress testing as enshrined in ICH guideline [1] and even this approach is being extended to drug combinations. This ICH guideline requires that stress testing on API and drug products should be carried out to establish their inherent stability characteristics, which should include the effect of temperature, humidity, light, and oxidizing agents as well as susceptibility across a wide range of pH. However, there are no detailed regulatory guidelines that direct how stress testing is to be done and hence stress testing has evolved into an “artful science” that is highly dependent on the experience of the pharmaceutical industries or the individuals directing the studies.

EMT, a synthetic nucleoside analog of cytidine, is phosphorylated by cellular enzymes to form EMT 5′-triphosphate. EMT 5′-triphosphate inhibits the activity of the HIV-1 RT by competing with the natural substrate deoxycytidine 5′-triphosphate and by being incorporated into nascent viral DNA which results in chain termination.

TDF is an orally bioavailable ester prodrug of tenofovir (also known as PMPA), an acyclic nucleotide analog with activity in vitro against retroviruses, including HIV-1, HIV-2, and hepatitis B virus (HBV). Due to the presence of a phosphonate group, TDF is negatively charged at neutral pH, which limits its oral bioavailability. Following absorption, TDF is rapidly converted to tenofovir which is metabolized intracellularly to the active metabolite, TDF diphosphate, a competitive inhibitor of HIV-1 RT that terminates the growing DNA chain. Acyclic phosphono-methyl ether nucleosides like TDF exhibit distinct biological properties. Due to their efficient intracellular activation to their active metabolites, they possess potent antiviral activity in vivo [2].

RPV is a second generation nonnucleoside reverse transcriptase inhibitor (NNRTI) for the treatment of HIV. It has higher potency, longer half-life, and reduced side-effect profile compared with older NNRTIs such as efavirenz. It has demonstrated activity against NNRTI resistant viral strains due to the flexibility of interactions with the HIV RT [3].

**Figure 1:**
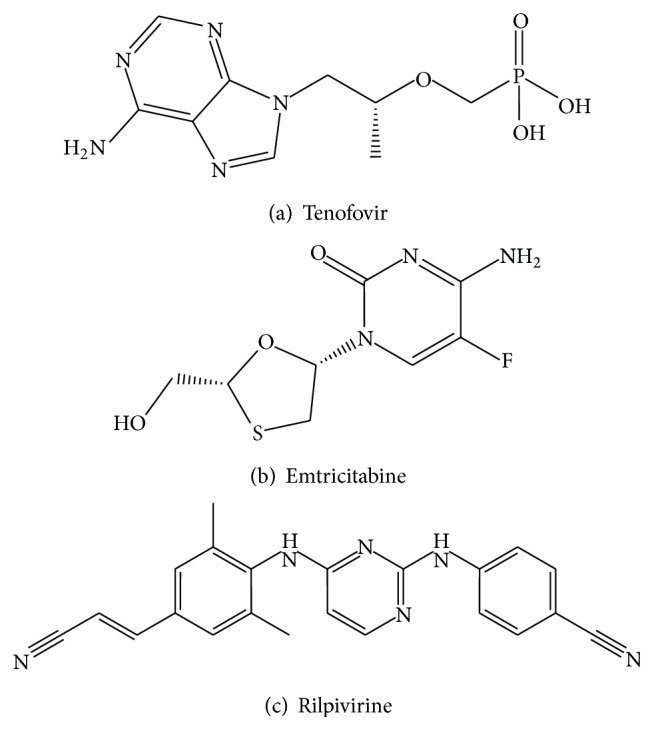
Chemical structure of analytes: (a) tenofovir, (b) emtricitabine, and (c) rilpivirine.

Highly active antiretroviral therapy (HAART) has brought new hope for those people who live with HIV/AIDS by decreasing the morbidity and mortality among people infected with HIV. Highly active antiretroviral therapy also has improved the quality of life among the people who live with HIV/AIDS [4]. The fixed dose combinations of nucleoside RT inhibitors EMT, TDF, and NNRTI RPV (Figure 1) are effective in the therapy of human immunodeficiency virus infection. It is used as a part of highly active antiretroviral therapy for the treatment of HIV. Three drug fixed dose combinations (FDCs) comprising EMT, TDF, and RPV form one of the first line regimens in HIV-therapy [5, 6].

Literature indicates that spectrophotometry [7–11], HPLC [12–15], HPTLC [16], and LC/MS/MS [17] methods for determination of TDF individually and in combination with other drugs in pharmaceutical formulations have been reported for drug substance and biological matrices.

Similarly, for EMT individually and in combination with other drugs by UV [18], HPLC in pharmaceutical formulations, drug substance, and biological matrices [19–21], LC/MS/MS [22], and stability-indicating liquid chromatographic methods [23] were reported.

A detailed literature survey for RPV revealed that few analytical methods are available using Spectrophotometry [24], HPLC [25] and HPTLC [26] were reported individually. However, an intensive literature search revealed to the best of our knowledge that only two methods are available for the determination of these analytes, EMT, TDF, and RPV, in pharmaceutical mixtures [27, 28].

Hence, recently, we have developed an optimized reversed-phase HPLC method for the routine quality control analysis of EMT, TDF, and RPV simultaneously from tablets. The method gave acceptable results for fresh quality control samples but gave overestimation during analysis of stability samples and aged products, as it lacks assay specificity in the presence of their degradation products. Further, no stability-indicating method has been reported in literature for simultaneous determination of EMT, TDF, and RPV in presence of their degradants.

Therefore, the present study targets the development and subsequent validation of a stability-indicating assay of new RP-HPLC method for the simultaneous determination of EMT, TDF, and RPV in presence of interaction/degradation product.

## 2. Material and Methods

### 2.1. Experimental

#### 2.1.1. Materials

Analytical pure samples of emtricitabine (C.C. Lab, Torrent Pharmaceutical Ltd., Indrad, India), tenofovir disoproxil fumarate (Sequent Research Lab., Mangalore, India), and rilpivirine (USV Ltd., Himachal Pradesh, India) were used in this study. The pharmaceutical dosage form used in this study was Complera procured from the local market and labelled to contain 200 mg of emtricitabine, 300 mg of tenofovir disoproxil fumarate, and 25 mg of rilpivirine per tablet. The solvent of acetonitrile (MeCN) was of HPLC grade and potassium dihydrogen phosphate, sodium hydroxide, hydrochloric acid, hydrogen peroxide, and triethylamine were of analytical reagent grade purchased from Merck, India. The HPLC grade water was prepared by using Milli-Q Academic, Millipore, Bangalore, India.

#### 2.1.2. Instrumentation

The LC system of Shimadzu model with UV-visible detector (SPD-10A) was used for this study and chromatographic separation was achieved on Phenomenex C18 (150 mm × 4.6 mm i.d., 5 *μ*m) column as stationary phase with isocratic mode.

#### 2.1.3. Chromatographic Condition

Chromatographic separations were carried out on a Phenomenex C18 analytical column (150 mm × 4.6 mm i.d., 5 *μ*m) connected with a Phenomenex C18 guard cartridge (4 mm × 3 mm i.d., 5 *μ*m). The mobile phase consisted of MeCN—potassium dihydrogen phosphate buffer (20 mM, pH 3.3)—and triethylamine. Wavelength of 270 nm was selected for detection. The injection volume of the sample was 20 *μ*L. The HPLC system was used in an air-conditioned laboratory atmosphere.

#### 2.1.4. Standard Solutions

Stock solutions of EMT, TDF, and RPV were prepared in mobile phase. Working standard solutions were freshly obtained by diluting the stock standard solutions with mobile phase during the day of analysis. The standard solution prepared for the optimization procedure constituted EMT, TDF, and RPV at 56.0, 84.0, and 7.0 *μ*g/mL, respectively.

#### 2.1.5. Sample Preparation

Twenty tablets were weighed and finely powdered. An amount of tablet powder equivalent to 20 mg of EMT and 30 mg of TDF with 2.5 mg of RPV was accurately weighed and transferred into a 50 mL volumetric flask. This mixture was subjected to sonication for 10 min for complete extraction of drugs. From the above solution, 7 mL of solution was pipetted out into 50 mL volumetric flask; the solution was made up to the mark with a mobile phase to obtain concentrations of 56.0, 84.0, and 7.0 *μ*g/mL of EMT, TDF, and RPV, respectively, for stability-indicating assay tablets.

#### 2.1.6. Forced Degradation Studies of API and Tablets

The marketed Complera tablets containing 200 mg of EMT, 300 mg of TDF, and 25 mg of RPV were subjected to various forced degradation conditions to effect partial degradation of the drug preferably in 2–30% range [29, 30]. The forced degradation studies were performed not only for the drug product, but also for API of EMT, TDF, and RPV to determine whether any observed degradation occurred because of drug properties or drug-excipient interactions. Moreover, the studies provide information about the conditions in which the drug is unstable so that measures can be taken during formulation to avoid potential instabilities. The stability samples were prepared by dissolving each API or drug product in mobile phase and later diluted with either distilled water, aqueous hydrochloric acid, aqueous sodium hydroxide, or aqueous hydrogen peroxide solution at concentrations of 400 (EMT), 600 (TDF), and 50 (RPV) *μ*g/mL separately. After the degradation treatments were completed, the stress content solutions were allowed to equilibrate to room temperature and dilute with mobile phase to achieve the nominal concentrations of 56 (EMT), 84 (TDF), and 7 (RPV) *μ*g/mL, which was based on their label strength in tablets.

#### 2.1.7. Acid Hydrolysis

Solutions for acid degradation studies were prepared in mobile phase and 0.3 N hydrochloric acid at room temperature for 24 hrs and mixture was neutralized and the resultant solutions were analyzed after 5–10 min of preparation.

#### 2.1.8. Base Hydrolysis

Solutions for base degradation studies were prepared in mobile phase and 0.1N sodium hydroxide at room temperature for 2 hrs and mixture was neutralized and the resultant solutions were analyzed after 5–10 min of preparation.

#### 2.1.9. Neutral Hydrolysis

Solutions for neutral degradation studies were prepared in mobile phase and water and the resultant solutions heated on a water bath at 90°C for 20 min. The mixture was neutralized and then allowed to cool at room temperature, filtered using syringe filters, and analyzed after 5–10 min of preparation.

#### 2.1.10. Oxidation Studies

Solutions for use in oxidation studies were prepared in mobile phase and 8% hydrogen peroxide at room temperature for 5 hrs and the resultant solutions were filtered using syringe filters and analyzed after 5–10 min.

#### 2.1.11. Photostability Studies

Solutions for photostability studies were prepared in mobile phase and the resultant solution was exposed to natural sunlight during the day time for 8 hrs for 3 days. The degraded sample was then filtered using syringe filters and analyzed.

#### 2.1.12. Temperature Stress Studies

Tablets and API in powder forms were exposed to dry heat (60°C) in an oven for 24 hrs. The API and tablet powders were then removed from the oven and an aliquot of tablet powder equivalent to the weight of one tablet was prepared for analysis as previously described.

## 3. Results and Discussion

### 3.1. HPLC Method Development

Our earlier HPLC method was optimized with respect to mobile phase composition, buffer concentration, and flow rate to achieve an optimal chromatographic condition for the separation and simultaneous quantification of EMT, TDF, and RPV from Complera tablet containing EMT 200 mg, TDF 300 mg, and RPV 25 mg. This optimized method employs Phenomenex Gemini C18 column (150 mm × 4.6 mm i.d., 5 *μ*m) and mobile phase consisting of MeCN—potassium dihydrogen phosphate buffer (20 mM, pH 3.3)—and triethylamine 58.72 : 41.23 : 0.05 (v/v) for the separation of EMT, TDF, and RPV without affecting the stability of these analytes. However, this method does not give data on specificity for the estimation of the three analytes in the presence of their degradants. Therefore, as an attempt to develop stability-indicating assay, the same optimal chromatographic condition has been tried to separate these analytes from the degradation products generated during forced degradation studies. Using this customized optimized method, it was possible to separate EMT, TDF, and RPV and their degradation products without any interference and thus the assay can be considered stability-indicating.

### 3.2. Degradation Behavior

Preliminary trials on individual drugs and those in tablet dosage form were conducted to optimize various stress conditions. Samples were withdrawn at 3–5 hr intervals to monitor the rate of degradation and optimize the stress conditions. Degradation conditions were attenuated such that all three drugs would get degraded in the range of 2–30% for establishing the stability-indicating nature of the assay method. Forced degradation studies of APIs of three analytes and formulations (Complera tablet) were carried out under various stress conditions.

**Figure 2:**
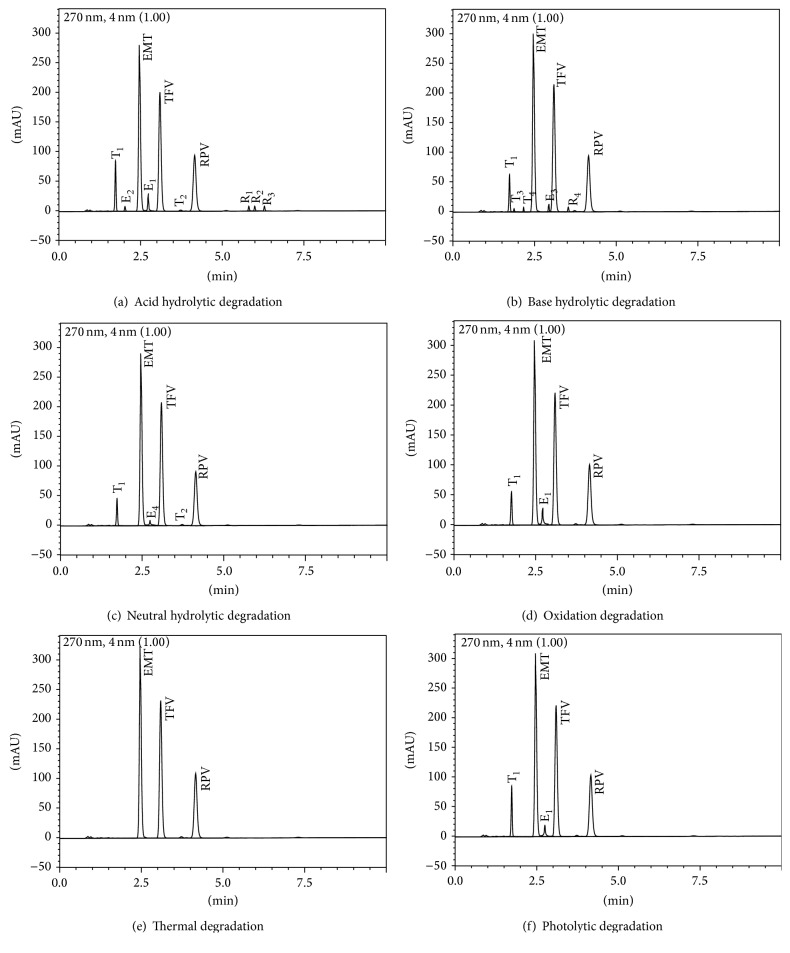
HPLC chromatographic separation of TDF, EMT, and RPV and its degradation product in different stress conditions.[The labeling of degradation products from its original source. For instance, emtricitabine (E_1_ E_2_ & E_3_), tenofovir (T_1_ T_2_ & T_3_) and rilpivirine (R_1_ R_2_ & R_3_)].

The extents of degradation of the three analytes in Complera tablet are shown in Figure 2. The degradation product formed from each drug has been identified by comparing the respective chromatograms of each API with formulations obtained after forced degradation studies. Each of the degradation or interaction products was given the name with respect to its original source like R_1_: the degradation product of RPV.

Labeling of all degradation products was done by a degrading individual drug with a similar condition as used for the combination tablets. Retention time and wavelength of degradation product were useful parameters to label degradation products. Such labeling was very useful to identify common degradation products among different degradation conditions.

All three APIs and tablet showed no degradation in 0.1 N HCl at room temperature for 24 hours; therefore, drastic condition was experimented with the use of higher concentration of HCl and reflux. EMT and TDF were easily susceptible to degradation in comparison to RPV in drastic condition and TDF and EMT undergo 18% and 16% decomposition under acidic stress condition for both pure and tablet dosage form. Similarly, RPV undergoes 3% decomposition under the same condition for both pure and tablet dosage form.

All drugs showed sufficient degradation when subjected to milder stress conditions of 0.1 N NaOH at room temperature for 2 hours. TDF was comparatively more prone to degradation under basic conditions. TDF underwent complete degradation when higher stress conditions were used. TDF undergoes 31% decomposition under basic stress condition whereas EMT and RPV undergo 17% and 7% degradation for both pure and tablet dosage form. RPV was quite stable under neutral degradation due to its nonpolar nature whereas TDF and EMT undergo 11% and 7% degradation, respectively, for both APIs and tablet dosage forms.

In oxidation stress condition, lower concentration of oxidizing agent was insufficient to give sufficient degradation for RPV even after prolonged exposure. Hence, samples were stressed at harsher conditions of 8% H_2_O_2 _at room temperature for 5 hrs. EMT, TDF, and RPV in APIs and tablets were moderately stable showing 12%, 13%, and 7% degradation, respectively.

In photolytic degradation, APIs and tablets were exposed to direct sunlight at 8 hours for 3 days. EMT, TDF, and RPV in APIs and tablets were highly stable and they undergo degradations of 2%, 6%, and 1%, respectively. Similarly, in thermal degradation subjected to dry heat (60°C) in an oven for 24 hours, EMT, TDF, and RPV in APIs and tablets were highly stable and they undergo degradations of 2%, 5%, and 1%, respectively. The degradation products of EMT, TDF, and RPV were found to be similar for all the formulations (Complera tablet) and API powders assessed.

The stability of stock solutions (stored at 2–8°C and at ambient temperature for 3 days) was determined by quantitation of each drug in solution in comparison to the response obtained for freshly prepared standard solutions. No significant changes (<2%) were observed for the chromatographic responses for the stock solutions analyzed, relative to freshly prepared standards.

The degradation study was not intended to identify the degradation products but merely to show that they would not interfere if and when they present. To conclude, the results of stress testing studies indicate a high degree of specificity of this method for EMT, TDF, and RPV.

### 3.3. Method Validation Parameters

The optimized HPLC method for the analysis of fresh quality control samples was validated in accordance with the ICH Q2 (R1) [31] guidelines and reported.

### 3.4. Selection of Wavelength

**Figure 3:**
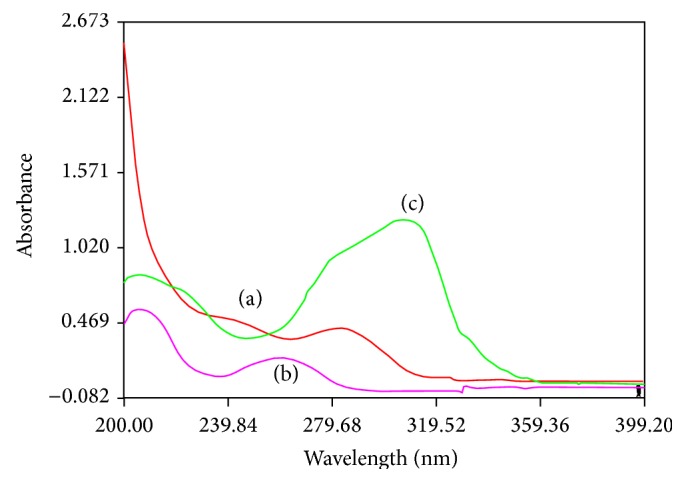
UV spectra of 10 *μ*g/mL each of EMT, TDF, and RPV in methanol. (a) UV spectrum of EMT; (b) UV spectrum of TDF; (c) UV spectrum of RPV.

The sensitivity of the HPLC method that uses UV detection depends upon the proper selection of the wavelength. The standard solutions were scanned from 200 to 400 nm and the overlain UV spectra obtained were recorded (Figure 3). From the overlain UV spectra, the detection wavelengths were selected for the methods to estimate simultaneously two or more drugs; the drugs in multicomponent dosage forms used in the present study gave good peak response at the wavelength selected.

### 3.5. Specificity

The results of forced degradation studies of each drug in the presence of their degradation products indicated a high degree of specificity of this method for EMT, TDF, and RPV. The degradation product of each of the parent peaks was found to be similar for the Complera tablet compared to that of API powders assessed. Typical chromatograms obtained following the assay of untreated and stressed samples of API and formulations are shown in Figure 2.

### 3.6. Accuracy

Accuracy of the method was determined by performing the recovery experiment at 50, 100, and 150% levels of the labeled number of the analytes in the commercial formulation. The recoveries for EMT, TDF, and RPV were found to be 99.50, 99.90, and 99%, respectively, which were within acceptable ranges of 100 ± 2%. The results are presented in Table 1.

### 3.7. Precision

The %RSD values for the intraday and interday precision were ≤2% confirming that the method was sufficiently precise. The results are presented in Table 2.

### 3.8. Linearity and Range

The linearity was established over the ranges of 28–84, 42–126, and 3.5–10.5 for EMT, TDF, and RPV, respectively. Correlation coefficients (*R* ^2^) were found to be more than 0.999 for all the analytes. Typically, the means of the regression equations were *y* = 87545*x* + 15731, *y* = 41493*x* + 18505, and *y* = 23045*x* + 65860 for EMT, TDF, and RPV. The LOD and LOQ were estimated as 1.90 and 0.43 ng/mL for EMT, 2.18 and 0.54 ng/mL for TDF, and 3.12 and 1.78 ng/mL for RPV.

### 3.9. Robustness Test

Robustness of the method was checked by small deliberate changes made in the method parameters such as wavelength (±2 nm), mobile phase ratio (±2%), flow rate (±0.1 mL), and pH (±0.05), but these changes did not affect the method results. The %RSD for the tablets were <2, indicating the robustness of the analytical methodology. The results are presented in Table 3.

### 3.10. Ruggedness

A study was conducted to determine the effect of variation in analyst to analyst, lab to lab, and instrument to instrument in triplicate measurement as per the assay method. %RSD was calculated for each condition and results are presented in Table 4.

### 3.11. Solution Stability Study

Table 5 shows the results obtained in the solution stability study at different time intervals for test preparation. It was concluded that the test preparation solution was found stable up to 72 h at 2–8°C and was at ambient temperature as during this time the result was not decreased below the minimum percentage.

### 3.12. Application of the Method

The optimized and validated formulation assay method was applied to the quantitative analysis of commercial tablet (Complera) and was evaluated by the proposed HPLC method. Good agreement was found between the assay results and the label claim of the product. The %RSD for the tablets were <2, indicating the precision of the analytical methodology. The results are presented in Table 6.

## 4. Conclusion

An isocratic stability-indicating HPLC-UV method has been developed for the estimation of EMT, TDF, and RPV in the presence of degradation products. The proposed method is simple, accurate, precise, and specific and has the ability to separate the drugs from degradation products and excipients found in the pharmaceutical dosage forms. The method is suitable for use in routine analysis of both drugs in bulk API powder or in pharmaceutical dosage forms. The method can be applied even to the analysis of stability samples obtained during accelerated stability experiments, as no interference was found with the degradants formed under various stress conditions.

## Figures and Tables

**Table 1:** Recovery studies for EMT, TDF, and RPV.

Con. (%)	Added amount (mg)			Amount recovered (mg)			Percentage recovered (%)		
	EMT	TDF	RPV	EMT	TDF	RPV	EMT	TDF	RPV
50	10	15	1.25	9.953	14.98	1.23	99.53	99.92	99.14
100	20	30	2.5	19.90	29.95	2.47	99.52	99.83	99.04
150	30	45	3.75	29.83	44.97	3.70	99.45	99.95	98.84

Mean % recovery (*n* = 9)							99.50	99.90	99.00
%RSD							0.044	0.104	0.061

**Table 2:** Precision results for EMT, TDF, and RPV.

Parameter	Sampling interval	EMT			TDF			RPV		
		Amount present (mg)	(%)	%RSD	Amount present (mg)	(%)	%RSD	Amount present (mg)	(%)	%RSD
Within-day	0 hrs	200.77	100.38	0.252	299.72	99.90	0.355	24.42	97.70	1.592
	8 hrs	198.69	99.34	0.100	299.48	99.82	0.487	24.74	98.99	0.181
	16 hrs	198.37	99.18	0.021	299.78	99.92	0.026	24.75	99.03	0.442

Between days	1st day	198.94	99.47	0.063	299.68	99.89	0.025	24.91	99.64	0.136
	2nd day	198.60	99.30	0.047	299.54	99.84	0.011	24.73	98.93	0.207
	3rd day	197.66	98.83	0.025	298.94	99.64	0.107	24.57	98.31	0.643

**Table 3:** Evaluation data of robustness study.

Parameter	Sampling interval	EMT			TDF			RPV		
		Amount present (mg)	(%)	%RSD	Amount present (mg)	(%)	%RSD	Amount present (mg)	(%)	%RSD
Wavelength	−1 nm	200.25	100.12	0.334	297.04	99.01	0.837	24.63	98.54	0.001
	+1 nm	200.10	100.50	0.624	299.47	99.82	0.323	24.92	99.68	0.029

Mobile phase	−2%	200.16	100.08	0.225	299.44	99.81	0.163	24.82	99.30	0.689
	+2%	200.09	100.04	0.430	298.92	99.64	0.509	24.68	98.75	0.370

Flow rate	−0.1 mL	198.81	99.40	0.837	300.55	100.18	0.019	24.47	97.89	1.469
	+0.1 mL	196.28	98.14	3.192	298.49	99.49	0.403	24.68	98.74	0.378

pH	−0.05	199.7	99.85	0.74	298.7	99.56	0.21	24.59	98.36	0.22
	+0.05	199.9	99.95	0.63	300.4	100.1	0.11	24.71	98.84	0.34

**Table 4:** Evaluation data of ruggedness study.

Parameter	EMT			TDF			RPV		
	(mg)	(%)	%RSD	(mg)	(%)	%RSD	(mg)	(%)	%RSD
Analyst 1	200.11	100.05	0.64	300.12	100.04	0.112	25.06	100.24	0.132
Analyst 2	200.62	100.31	0.33	298.94	99.64	0.145	24.79	99.16	1.232
Instrument 1	200.18	100.09	0.634	300.29	100.09	0.146	24.81	99.26	0.246
Instrument 2	200.32	100.16	0.357	299.15	99.71	1.133	24.81	99.26	0.241
Lab 1	200.21	100.10	0.470	306.45	102.15	1.059	25.32	101.29	1.976
Lab 2	199.58	99.79	0.354	298.96	99.65	0.103	24.64	98.57	0.103

**Table 5:** Evaluation data of solution stability study.

Day	EMT	TDF	RPV	EMT	TDF	RPV
	% assay for test preparation solution at 2–8°C			% assay for test preparation solution at ambient temperature		
Initial	99.96	99.89	99.89	99.82	100.20	99.16
1	99.89	99.75	99.84	99.82	100.92	99.17
2	99.92	98.79	99.77	98.24	99.24	98.91
3	98.45	98.59	99.56	98.45	99.09	98.44

**Table 6:** Assay results for commercial formulation.

Amount present (mg)	(%)	Amount present (mg)	(%)	Amount present (mg)	(%)
EMT		TDF		RPV	
198.21	99.10	300.48	100.16	24.92	99.70
199.07	99.53	299.81	99.93	24.91	99.65
198.21	99.10	296.63	98.87	24.63	98.54
201.12	100.56	299.57	99.85	24.92	99.70
200.55	100.27	298.91	99.63	24.94	99.77
199.92	99.96	300.48	100.16	24.91	99.65

S.D	0.579	S.D	0.494	S.D	0.523
%RSD	0.579	%RSD	0.495	%RSD	0.525
